# GROWING UP ON A FARM AND COGNITIVE FUNCTIONING IN LATER LIFE

**DOI:** 10.1093/geroni/igz038.221

**Published:** 2019-11-08

**Authors:** Pamela Herd, Sanjay Asthana, Kamil Sicinski

**Affiliations:** 1 Georgetown University, Washington, District of Columbia, United States; 2 UW-Madison, Madison, Wisconsin, United States

## Abstract

There is growing interest in rural disadvantage and the implications for health and well-being in later life. We examine the relationship between living in rural areas in childhood and cognitive outcomes later in life using the Wisconsin Longitudinal Study. The WLS has prospective childhood measures of geographic status, adolescent IQ, and detailed measures of socioeconomic status, combined with later life measures of health and cognitive functioning. We find a robust relationship between rurality and lower levels of cognitive functioning, but it is explained by growing up on a farm.

